# Plasmid prevalence is independent of antibiotic resistance in environmental Enterobacteriaceae

**DOI:** 10.1099/mgen.0.001453

**Published:** 2025-08-12

**Authors:** Danya Gewurz, Suhyeon Kim, Lorenza Bartu, Abhishek Sharma, Joanna Ciol Harrison, Ivan Lee, Nicole C. Rondeau, JJ L. Miranda, Brian J. Mailloux, Kerry A. Hamilton, Allison J. Lopatkin

**Affiliations:** 1 Department of Biology, Barnard College of Columbia University, New York, NY, USA; 2 Department of Chemical Engineering, University of Rochester, Rochester, NY, USA; 3 Department of immunology and Immunotherapy, Icahn School of Medicine at Mount Sinai, New York, NY, USA; 4 Goergen Institute for Data Science and Artificial Intelligence, University of Rocehster, Rocehster, NY, USA; 5 School of Sustainable Engineering and the Built Environment, 660 S College Ave, Tempe, AZ, USA; 6 The Biodesign Center for Environmental Health Engineering, 1001 S McAllister Ave, Tempe, AZ, USA; 7 Department of Environmental Sciences, Barnard College of Columbia University, New York, NY, USA; 8 Department of Microbiology & Immunology, University of Rochester Medical Center, Rochester, NY, USA; 9 Department of Biomedical Engineering, University of Rochester Medical Center, Rochester, NY, USA

**Keywords:** antibiotic resistance, horizontal gene transfer, plasmid

## Abstract

The rapid rise of antibiotic-resistant pathogens poses a critical threat to the treatment of infectious diseases. While the spread of antibiotic resistance genes (ARGs) via plasmid conjugation has been extensively studied both in the lab and the clinic, the prevalence and diversity of plasmids in drug-susceptible isolates (e.g. isolates that do not contain ARGs) remain poorly understood. Yet, plasmids in susceptible isolates play a pivotal role as reservoirs, potentially capturing and disseminating ARGs *in situ*. To better understand the potential impact of these strains, we investigated the prevalence and characteristics of plasmids in >200 *Enterobacteriaceae*, including those that are primarily drug susceptible, isolated from diverse environmental sources. Using whole-genome sequencing and a novel bioinformatic pipeline, we quantified the number of large plasmids per isolate and examined the relationship between plasmid abundance and host antibiotic susceptibility profiles. Strikingly, we found a high abundance of plasmids in susceptible strains, with no correlation between plasmid number and susceptibility level to a variety of clinically relevant antibiotics. Moreover, plasmid abundance did not influence a strain’s ability to accept additional plasmids via conjugation. These findings reveal that plasmids are widespread in susceptible strains regardless of ARG content and underscore their potential to act as conduits for future resistance dissemination.

**Impact Statement**

The evolution of antibiotic resistance is frequently linked to plasmid-mediated horizontal gene transfer, yet the role of plasmids in primarily drug-susceptible bacterial populations remains poorly understood. Here, we show that plasmids are equally abundant in susceptible and resistant *Enterobacteriaceae* isolates, challenging the assumption that plasmid prevalence is driven by antibiotic selection. Moreover, we demonstrate that the presence of pre-existing plasmids does not restrict conjugation efficiency, highlighting the potential of susceptible strains as reservoirs for future resistance dissemination. These findings underscore the importance of integrating plasmid surveillance in environmental and clinical microbial studies and provide new insights into the broader ecology of antibiotic resistance evolution.

## Data Summary

All 205 raw short-read and 33 long-read sequencing data are deposited on NCBI under the BioProject Accession ID PRJNA1190092. Data corresponding to Table S5 can be found at the following URL: https://figshare.com/articles/dataset/TableS5_Dataset/29282048/1. All other raw data are provided in the supplementary information.

## Introduction

Horizontal gene transfer (HGT), which describes the movement of genetic material between organisms, is a major contributor to the dissemination of antibiotic resistance genes (ARGs). Although HGT occurs by several mechanisms, plasmid conjugation – the transfer of plasmid DNA via direct cell-to-cell contact – is considered the primary mechanism by which ARGs spread [1,2]. Indeed, previous analyses have shown that plasmids can carry genes that confer resistance to all available antibiotics [3]. These ARG-carrying plasmids often coexist by the thousands in dense diverse microbiomes that act as resistance reservoirs; once present, opportunistic pathogens and commensal microbes can rapidly acquire new ARGs and other virulence factors via HGT [4,6]. Additionally, the proximity of diverse plasmids increases the likelihood of genetic exchange or acquisition by individual plasmids. Thus, establishing the factors mediating plasmid and ARG dispersal is critical to predicting and mitigating the further rise of resistant pathogens.

Historically, plasmids have been classified based on their ability to stably coexist within the same bacterial host, a property known as incompatibility (Inc) [7,9]; plasmids that are incompatible with each other are assigned to the same Inc group. Although this system has some limitations and several alternatives have been developed [10,11], extensive surveillance efforts have revealed clear associations between certain Inc groups and ARG dissemination patterns. Amongst *Enterobacteriaceae* in particular, some Inc groups have emerged as epidemic (e.g. IncF, IncI, IncA/C and IncH) [12,14] due to their association with specific ARGs [15]. These strong patterns have led to the well-established observation that plasmids are often highly prevalent in drug-resistant *Enterobacteriaceae* strains [15,16]. However, far less is known about plasmids in primarily drug-susceptible isolates. Specifically, drug-susceptible isolates may truly harbour fewer plasmids overall, or those that they harbour encode fewer ARGs. Resolving this uncertainty is critical, as plasmids in susceptible isolates have the capacity to acquire ARGs (e.g. through transposition) or influence the acquisition of additional plasmids by their host (e.g. through Inc). Thus, despite not being direct determinants of AMR, these plasmids could play an underappreciated role in resistance propagation.

To better understand the relationship between plasmid abundance and drug susceptibility phenotypes, here, we collected and deeply characterized a library of over 200 environmental strains with a diverse range of susceptibility levels, isolated from New York City and throughout the Midwest. We then assessed the role of susceptible isolates in the spread of ARGs by quantifying how pre-existing plasmid number, regardless of exclusion mechanisms, impacts subsequent conjugation capabilities.

## Methods

### Collecting environmental isolates from New York City and the US Midwest

Wastewater was collected from a primary academic building at Barnard College in New York City and stored at 4 °C for up to 24 h. To isolate *Enterobacteriaceae*, 10 mL of 99 : 1 diluted wastewater was spread onto an Eosin Methylene Blue (EMB) agar plate and incubated at 37 °C for 16 h. Single colonies, with preference to those that appeared neon green to prioritize *Escherichia coli*, were then streaked onto a fresh EMB agar plate and grown under the same condition. Single colonies were inoculated into Lysogeny Broth (LB) media and cultured at 37 °C with shaking at 250 r.p.m. for 16 h. Cultures were preserved in 25% glycerol at −80 °C. From the 205 total isolates, 100 of them (denoted AL) were randomly selected for subsequent analysis. An additional 105 isolates (denoted KH) were collected from diverse locations across the USA, including the Midwest, South and West (Table S1, available in the online Supplementary Material). Samples were collected in sterile polypropylene bottles and either processed immediately or frozen for transport, then processed within 24 h. A 45–50 mL aliquot was diluted in phosphate-buffered saline and vacuum-filtered through a 0.45 µm white-gridded membrane filter (Merck Millipore, Burlington, MA). The filter was placed on an mTEC agar culture plate, a medium used for chromogenic detection and incubated overnight at 37 °C. Red or magenta colonies were considered *E. coli* isolates and were picked using a sterile toothpick, cultured in LB media for 16 h with shaking at 250 r.p.m. and preserved in 25% glycerol at −80 °C. Detailed information on all isolates is provided in Tables S1–S3.

### Susceptibility testing

Antibiotic susceptibility testing was performed for each strain using Sensititre^™^ Gram Negative MIC Plates (Thermo Fisher, Catalogue Number GN3F), following the manufacturer’s instructions with the several modifications: overnight cultures of purified colonies were grown in LB at 37 °C with shaking at 250 r.p.m. for 16 h. Each strain was then diluted 1 : 10,000 in Mueller–Hinton media, and 50 µL of diluted cells was pipetted into every well of a Sensititre plate. Plates were grown for 24 h in a 37 °C incubator and then scanned on a flatbed Epson v800 scanner to automate quantification. The images were processed using a custom MATLAB image analysis suite to determine the wells that exhibited growth. The MIC was determined as the lowest antibiotic concentration that entirely inhibited growth, and strains were classified as S, I or R based on the MIC according to clinical standards (Table S4). In all cases, positive and negative control wells were used to confirm the analysis validity.

### Whole-genome sequencing and assembly

DNA was extracted from overnight cultures of each colony using the PureLink Genomic Mini Kit (Invitrogen^™^, New York, NY), following the manufacturer’s instructions. A plexWell LP384 Library Preparation Kit was used to prepare samples for sequencing (SeqWell, Beverly, MA). Quality control (QC) was performed on a tape station prior to sequencing on a MiSeq instrument with 2×250 bp reads and a desired fragment size of 398 bp (~30–40× coverage in total targeting a 4.6 mb genome). Thirty-three strains were randomly chosen for sequencing using PacBio (CGGENOMICS). To assemble short-read Illumina data, raw reads were first demultiplexed, trimmed using Trimmomatic [17] and assembled using SPAdes [18] with default parameters. Hybrid assemblies were generated for any strain that had accompanying long-read sequencing using UniCycler [19] instead of SPAdes. We then used PlasmidFinder [20] to identify all contigs from assembled genomes with an identifiable replicon and at least 95 % identity to any plasmid found on the PLSDB database [21]. A consensus between ResFinder, CARD database (https://card.mcmaster.ca/) and AMRfinder tools was used to verify the presence of ARGs on all putative plasmid contigs. All assemblies were annotated according to their phylotype and sequence type if known, and a consensus genus was determined using Kraken [22] and kmerfinder [23]. All genomic metadata are found in Table S1. Although 205 isolates were initially sequenced, one strain (KH_23) failed to regrow for follow-up analysis. Therefore, all downstream analyses were performed on the remaining 204 isolates.

### Counting the number of plasmids

To count plasmids, a comprehensive pipeline was developed with the following key steps: (1) plasmid identification with PlasmidFinder: FASTA files were processed using the PlasmidFinder [20] tool. Results were filtered based on coverage (≥80%), identity (≥90 %) and read length thresholds to ensure accuracy. (2) Data filtering and preprocessing: duplicate entries and low-quality plasmid predictions were removed based on predefined criteria, including minimal contig length (≥500 bp), and sufficient coverage (>3×). (3) Collapsing and de-duplicating contigs: replicons found on the same contig were combined into a single entry representing a single putative plasmid (i.e. contig) containing multiple replicons, and plasmids with overlapping sequences, determined using Mash distance analysis, were combined into unified entries. This step ensures unique and accurate representation of plasmid sequences. (4) Validation with PLSdb blast: remaining plasmid contigs were blasted against the PLSdb database to validate their identity. Only hits exceeding specified thresholds for identity (≥90%) and length (≥1,000 bp) were retained. Contigs with significant overlap (≥90%) were further collapsed into single plasmid entries. (5) Quantification of plasmids per sample: final contigs were grouped by sample, and the number of unique plasmids was tallied for each strain. Samples without plasmid predictions were explicitly recorded with a count of zero. (6) Output generation: two summary outputs were generated: (i) a detailed file listing all identified plasmids, including contig information, plasmid names and blast results; (ii) an aggregated file providing the total plasmid count per sample for downstream analyses. All code is freely available under https://github.com/ajlopatkin/plasmidcounts, plasmidcounts-v1.0.0 release. For completeness, details on every plasmid identified in this dataset can be found in Table S5. Plasmids included for subsequent analysis were those present at a coverage of 1.5× or greater; this filter did not change any main conclusions.

### PlasmidCounts validation and calibration

To assess the need for long-read data in identifying appropriate parameter sets, we validated the PlasmidCounts pipeline using both sensitivity and bootstrapping analyses on our own genome sequences, along with publicly available completed genome sequences. First, we conducted a coarse sensitivity analysis across a wide range of values for seven key parameters, along with the presence/absence of small ‘col’ replicons, showing that mismatched plasmid counts remained largely low across broad parameter regimes (Fig. S1). We then defined refined parameter ranges (±75% around base values) and randomly sampled from these ranges to generate 100 unique parameter sets. We note that the parameters varied are those included in Fig. S1, excluding the binary ‘col’ filter, which was left on. These were used in a bootstrap calibration procedure, where subsets of n strains (from 3 to 28) were randomly sampled from the set of 33 genomes with paired long-read data. Calibration success was defined as any parameter set achieving >90% concordance between predicted (from short reads) and true (from long reads) plasmid counts. We found that ~20 strains were sufficient to achieve stable, high-confidence calibration, yielding >95% accuracy across all parameters, with little improvement beyond this point (Fig. S2A). To further validate performance, we tested the calibrated parameters on 23 completed *E. coli* genomes previously analysed (Table S6) [24]. We simulated 250 bp paired-end reads at 100× coverage using InSilicoSeq [25] and assembled each genome using our standard pipeline. PlasmidCounts then predicted plasmid content from the assemblies. As shown in Fig. S2B, using the base parameters and 100 randomly sampled parameter sets (±75% variation), we recovered ~80% of known plasmid replicons using a subset of *n*=12 randomly sampled strains (from those listed in Table S6), with >93% accuracy in plasmid counts, even without further calibration.

### Evolving rifampicin-resistant recipients

To enable selection of transconjugants via rifampicin resistance, rifampicin-resistant mutants were evolved from each recipient strain. Strains were grown overnight and concentrated tenfold in LB. Fifty microlitres were then spread onto rifampicin agar plates and placed in a 37 °C incubator to grow for 20–24 h. Single colonies were then picked from the plate and regrown in LB mixed with rifampicin for 16 h in a 37 °C incubator at 250 r.p.m. Each strain was stored at −80 °C in 25% glycerol.

### Quantifying conjugation efficiencies

Conjugation assays were performed following our previously established protocols [26,27]. Donor strains [*E. coli* carrying pCDC-61 (gentamicin resistance) or pOX38 (tetracycline resistance)] and rifampicin-resistant recipients were grown overnight in LB media. Both were resuspended in M9CAG media at a 1 : 1 ratio. Parents were allowed to rest for 5 min and then mixed in a 1 : 1 ratio and incubated at 25 °C for 1 h without shaking. After the 1 h incubation, mixtures were diluted serially, and 20 µL of the transconjugants was spot-plated onto three antibiotic plates corresponding to the three populations (e.g. gentamicin, rifampicin and gentamicin-rifampicin). These plates were grown for 24 h at 30 °C. Donor, recipient and transconjugant colony forming units (c.f.u). were then counted on respective single and double antibiotic plates. The conjugation efficiency (η) was calculated using the formula:


$$\eta =\frac{T}{RD\Delta t}$$

where *T* is the number of transconjugant colonies, *R* is the number of recipient c.f.u., *D* is the number of donor c.f.u. and Δt is the time of conjugation in hour.

## Results

After collecting 205 *Enterobacteriaceae*, we measured each strain’s MIC against [4] panel of 21 common Gram-negative antibiotics spanning several representative mechanisms of action. Clinical breakpoints were used to assign ‘Susceptible’ (S), ‘Intermediate’ (I) or ‘Resistant’ (R) designations for each strain-drug pair (Table S4). To evaluate overall susceptibility, we grouped S and I, and R, and defined the non-resistant (NR) percentage over all 21 antibiotics as follows:


$$\%NR=100*\frac{S+I}{S+I+R}$$

**Fig. 1.**
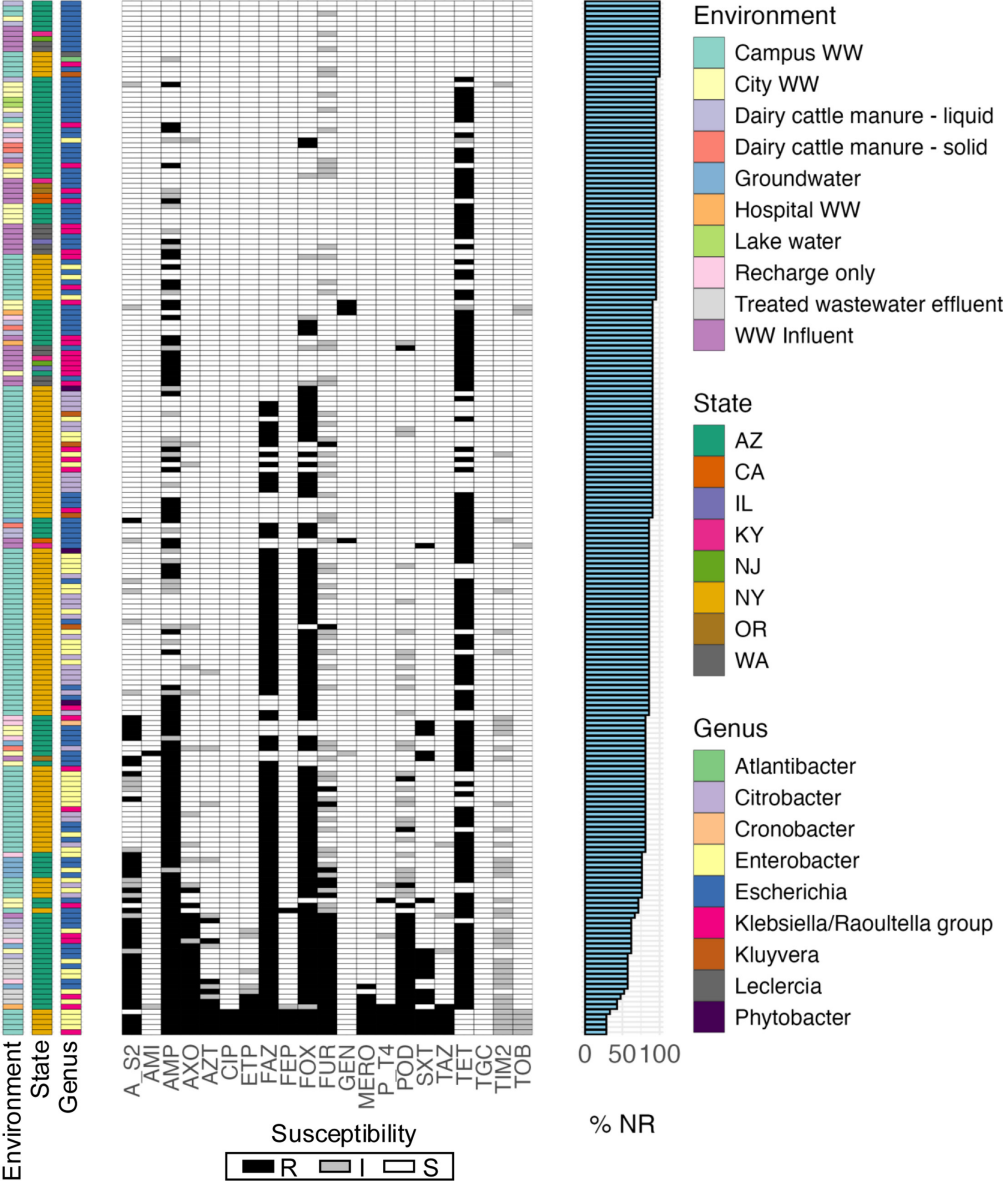
Summary of *Enterobacteriaceae* isolates with a range of susceptibility levels. Metadata and susceptibility information for 205 *Enterobacteriaceae* isolates collected from various environmental sources. Single-bar heat maps from left to right indicate the environment each isolate was collected from, the geographic state and the sequenced-verified genus. The middle heat map indicates resistance (black) or non-resistance (susceptible or intermediate, white) for all 21 antibiotics tested; antibiotic abbreviations are amikacin (AMI), ampicillin (AMP), ampicillin/sulbactam (A/S2), aztreonam (AZT), cefazolin (FAZ), cefepime (FEP), cephalothin (CEP), meropenem (MERO), ertapenem (ETP), cefuroxime (FUR), gentamicin (GEN), ciprofloxacin (CIP), piperacillin/tazobactam (P/T4), cefoxitin (FOX), trimethoprim/sulphamethoxazole (SXT), cefpodoxime (POD), ceftazidime (TAZ), tobramycin (TOB), tigecycline (TGC), ticarcillin/clavulanic acid (TIM2), ceftriaxone (AXO) and tetracycline (TET). The right-most bar graph shows the NR percentage for each isolate across all 21 antibiotics.

Strains ranged from 28.6% to 100% NR, with a median of 88.1% (Fig. 1, Table S1). This indicates that most strains were resistant to two or fewer antibiotics, and no strains were resistant to >15 antibiotics. We next applied whole-genome Illumina sequencing on all strains; annotations confirmed the isolates consisted of *Enterobacteriaceae* from various genera (Fig. 1).

To determine plasmid prevalence, we developed a novel informatics tool, PlasmidCounts, which estimates the number of unique large plasmids (>1,000 bp, excluding small Col-type replicons) per isolate in short-read datasets, using calibration against a small number of paired long-read genomes and integration with published plasmid sequences (see Methods); this plasmid size threshold emphasizes those most relevant for conjugation and ARG presence. Independent validation using high-coverage long-read assemblies for 33 randomly selected strains confirmed that PlasmidCounts accurately counts the total number of unique large plasmids (Fig. S1). Systematic sensitivity analysis confirmed that long-read pairing for only ~10% of the dataset was sufficient for calibration; 20 out of 205 genomes enabled >95% average replicon match across 100 randomly sampled parameter sets, with the best-performing set achieving 100% accurate plasmid counts (Fig. S2A).

**Fig. 2.**
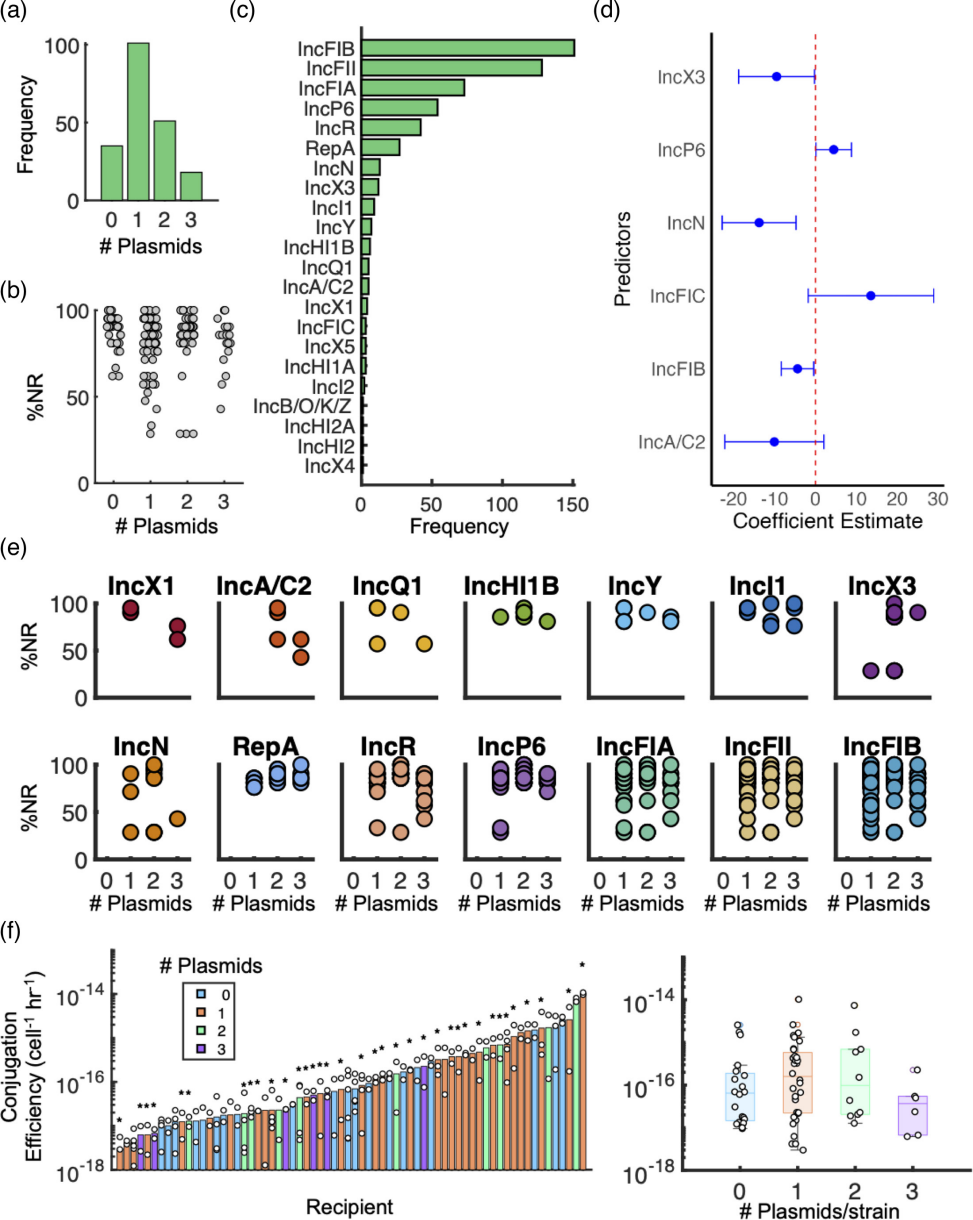
No significant relationships between plasmid count and strain phenotypes. (a) Distribution of the number of large plasmids per strain for all 205 isolates. (b) Susceptibility phenotype, defined by the percentage of non-resistance, is uncorrelated with the number of plasmids per strain. (c) Distribution of Inc groups in the strain library. (d) Estimated coefficients (estimates) representing the relationship between susceptibility and prominent Inc groups, as determined by the stepwise regression model; error bars indicate 95% CI. IncN is the only significant covariate with an adjusted *P*-value of 0.018. (e) NR percentage plotted by the number of plasmids per strain, split up by Inc group. (f) *Left*: conjugation efficiencies for a subset of 68 strains; bars are coloured by the number of plasmids per strain. Star markers indicate the presence of a pre-existing IncF plasmid in the strain. *Right*: box plot of data on the left; no statistical difference between conjugation efficiencies as a function of the number of plasmids carried by the recipient. Bottom and top whiskers indicate the fifth and ninety-fifth percentiles, respectively.

Out of 205 strains, 137 carried 0 or 1 plasmid, whereas 68 carried 2 or 3 plasmids (Fig. 2). We found no statistical relationship between plasmid count and susceptibility percentage (one-way ANOVA, *P*=0.4659, R²=0.004674, Fig. 2), indicating that the number of plasmids a strain carried had no bearing on its susceptibility phenotype. We also compared these distributions to plasmid counts from our recently assembled dataset of *E. coli* genomes downloaded from NCBI with associated completed plasmids [24]. Excluding those with no plasmid (which were not included in that dataset), the distributions of plasmid frequencies were statistically equivalent (*P*=0.8965, Wilcoxon rank sum test).

Across all strains, there were 23 distinct Inc groups, ranging in frequency from 1 to 219 (Fig. 2); the IncF family was most abundant (IncFII, IncFIB and IncFIA), consistent with its prevalence in this taxon group when associated with ARGs [13,28]. However, NR percentages in the 155 IncF-carrying isolates still ranged from 28% to 100%, and the mean %NR did not significantly change (*P*=0.5638, permutation test). Indeed, further multivariate regression analysis revealed that most Inc group identities, more generally, were not predictive of %NR (Fig. 2), consistent with qualitative inspection (Fig. 2), although some trends were observed. Of all Inc groups, IncN is the only one significantly correlated with %NR (*P*=0.018, multiple linear regression).

Finally, to determine whether the presence of a pre-existing plasmid(s) impacts a strain’s ability to accept a new plasmid, we quantified the conjugation efficiency for 68 strains covering 6 genera, with a focus on *E. coli* (Fig. S3A). We used the IncF pOX38 conjugative plasmid, which would be most likely to exhibit exclusion given the pre-existing IncF prevalence. Conjugation efficiencies ranged widely (Figs 2 and S3B), with no association between pre-existing plasmid count (Fig. 2) or susceptibility level (Fig. S3C) (ρ=0.02, Spearman correlation coefficient). To confirm that this result was not plasmid-specific, we selected a subset of 12 strains, 6 each with the minimum (0) and maximum (3) number of plasmids, and compared their conjugation efficiencies using a second clinically relevant plasmid (pCDC-61, IncF/N) obtained from the CDC. Results confirmed no association between pre-existing plasmids and conjugation efficiency, consistent with pOX38 (Fig. S3D).

## Discussion

Extensive research has been conducted on the diversity and prevalence of plasmids associated with antibiotic resistance determinants [12,14, 29,31]. In contrast, we focused on plasmids associated with drug-susceptible phenotypes due to their relevance as the evolutionary precursor to resistance acquisition. To assess the prevalence of plasmids in these isolates, we collected 205 *Enterobacteriaceae* and categorized each strain’s NR percentage. Using whole-genome sequencing, we were able to confirm that our dataset is representative of other *Enterobacteriaceae* genomes. Additionally, we determined the number of plasmids within each of the strains and discovered that plasmids are equivalently abundant in primarily susceptible and non-susceptible isolates. Further, PlasmidCounts enables users to quantify unique large plasmids per strain from short-read data, requiring only minimal long-read input for calibration. The accuracy of these counts is expected to vary depending on dataset-specific factors such as sequencing method, coverage depth and strain composition. In our dataset, calibration with ~20 long-read-paired strains was sufficient to achieve 100% accurate plasmid counts. In contrast, applying the default parameters to simulated short-read data recovered ~80% of known replicons on average, with 93% accuracy in plasmid counts. We expect that broader parameter sampling will enable users to achieve similarly high accuracy on their own datasets.

Surprisingly, the IncF family of plasmids, which is previously known to be associated with ARGs, is similarly abundant across susceptibility levels, including strains without ARGs. These findings reveal that large plasmids are similarly prevalent in environmental *Enterobacteriaceae* regardless of drug susceptibility levels – counterintuitively, they are not restricted to or biassed towards antibiotic-resistant strains. Recent work highlights the role of ARG-encoding plasmids as highly mobile and often associated with broad host ranges, particularly in multidrug-resistant strains [32]. Our study complements this view by showing that large plasmids, regardless of ARG content, are also prevalent in susceptible populations. This suggests that ARG-encoding plasmids may represent a specialized subset of a much broader and ecologically embedded plasmid pool contributing to the spread of resistance.

Intuitively, having more resident conjugative plasmids in a cell may reduce the ability of a strain to accept another plasmid, due in part to entry exclusion and plasmid Inc mechanisms. To further investigate this, we used conjugation efficiency experiments with a panel of *E. coli* recipients. *E. coli* was chosen as our sample species because it is an effective mediator of transferable resistance between bacteria, exhibits a wide range of genomic variability and was the most abundant species in our dataset. However, conjugation experiments showed no association between pre-existing plasmid numbers and the ability to acquire new plasmids, which underscores the role drug-susceptible hosts may play in accepting new antibiotic resistance. Moreover, the observation that pre-existing IncF plasmids do not significantly interfere with conjugation efficiency highlights that the two may not be as incompatible as previously thought and aligns with emerging views that traditional definitions of plasmid incompatibility do not fully capture the complexity of plasmid–plasmid interactions [8]. Overall, this work lays a foundation for tracking the evolution of strains from susceptibility to resistance and offers the community a fully sequenced, phenotypically characterized set of drug-susceptible *Enterobacteriaceae* strains for subsequent ecological and evolutionary studies.

## Supplementary material

10.1099/mgen.0.001453 Uncited Supplementary Material 1.

10.1099/mgen.0.001453 Uncited Supplementary Material 2.

10.1099/mgen.0.001453 Uncited Supplementary Material 3.

10.1099/mgen.0.001453 Uncited Supplementary Material 4.

## Abbreviations

ARGs: Antibiotic Resistance Genes
EMB: Eosin Methylene Blue
HGT: Horizontal Gene Transfer
Inc: INcompatibility
LB: Lysogeny Broth
NR: Non-resistant
