# Supplementary material for: Plasmid prevalence is independent of antibiotic resistance in environmental Enterobacteriaceae
Source: Microb Genom. 2025 Aug 12;11(8):001453. doi: 10.1099/mgen.0.001453 (PMC12452199; doi:10.1099/mgen.0.001453)
Supplement: Uncited Supplementary Material 1. [file mgen-11-01453-s001.pdf]

Supplementary information for:

Plasmid prevalence is independent of antibiotic resistance in environmental *Enterobacteriaceae*

Danya Gewurz[1,+], Suhyeon Kim[2,+], Lorenza Bartu[3], Abhishek Sharma[2,4], Johanna Ciol Harrison[5,6], Ivan Lee[2], Nicole C. Rondeau[1], JJ L. Miranda[1], Brian J. Mailloux[7], Kerry A. Hamilton[5,6], and Allison J. Lopatkin[2,8,9,*]

[1]Department of Biology, Barnard College of Columbia University, New York NY
[2]Department of Chemical Engineering, University of Rochester, Rochester NY
[3]Department of Genetics and Genomics, Mt. Sinai School of Medicine, New York NY
[4]Department of Data Science, University of Rochester, Rochester NY
[5]School of Sustainable Engineering and the Built Environment, 660 S College Ave, Tempe AZ
[6]The Biodesign Center for Environmental Health Engineering, 1001 S McAllister Ave, Tempe AZ
[7]Department of Environmental Sciences, Barnard College of Columbia University, New York NY
[8]Department of Microbiology & Immunology, University of Rochester Medical Center, Rochester NY
[9]Department of Biomedical Engineering, University of Rochester Medical Center, Rochester NY
* Corresponding author: allison.lopatkin@rochester.edu
[+] These authors contributed equally to this work

Supplementary Figures S1-S3
Supplementary Tables S1-S6

Supplementary Figures:

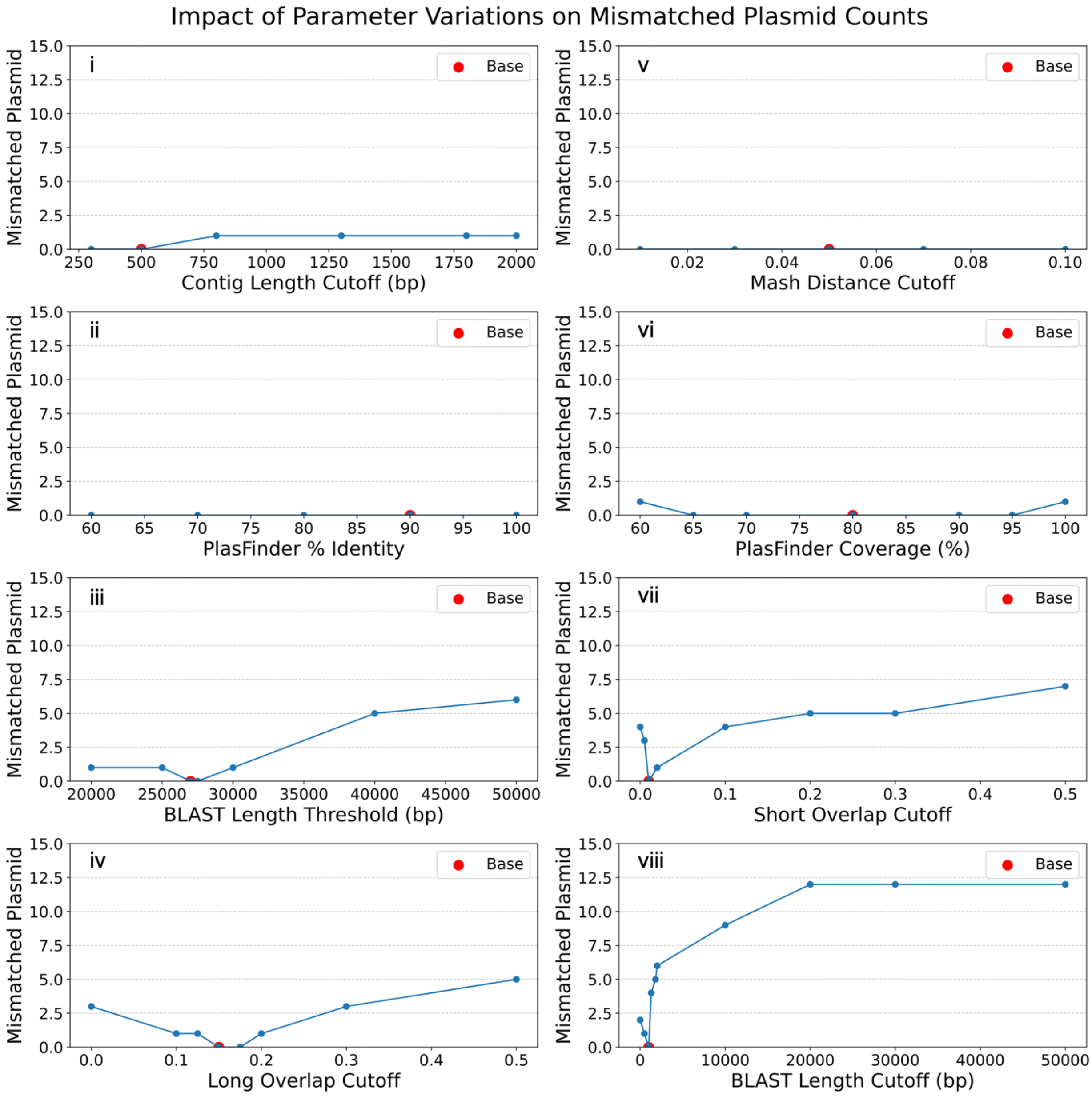

Figure S1: PlasmidCounts sensitivity analysis. The PlasmidCounts pipeline was run on the 33 genomes assembled using either short or long-read sequencing. The plasmid count was determined from both assemblies, and the number of mismatches calculated by subtracting the difference (e.g., (abs(#shortread_plasmid - #longread_plasmid)). No mismatches (y-axis) were found using the base model parameters (red circle). A sensitivity analysis was conducted for all main parameters by varying each main parameter one at a time, and plasmid mismatches were calculated in each case. Each panel shows the effect for a distinct parameter: i) contig length cutoff, (ii) %Identity for plasmidfinder, (iii) BLAST length threshold, (iv) long overlap cutoff, (v) MASH distance, (vi) plasmidfinder % coverage, vii) short overlap cutoff, and viii) BLAST length cutoff.

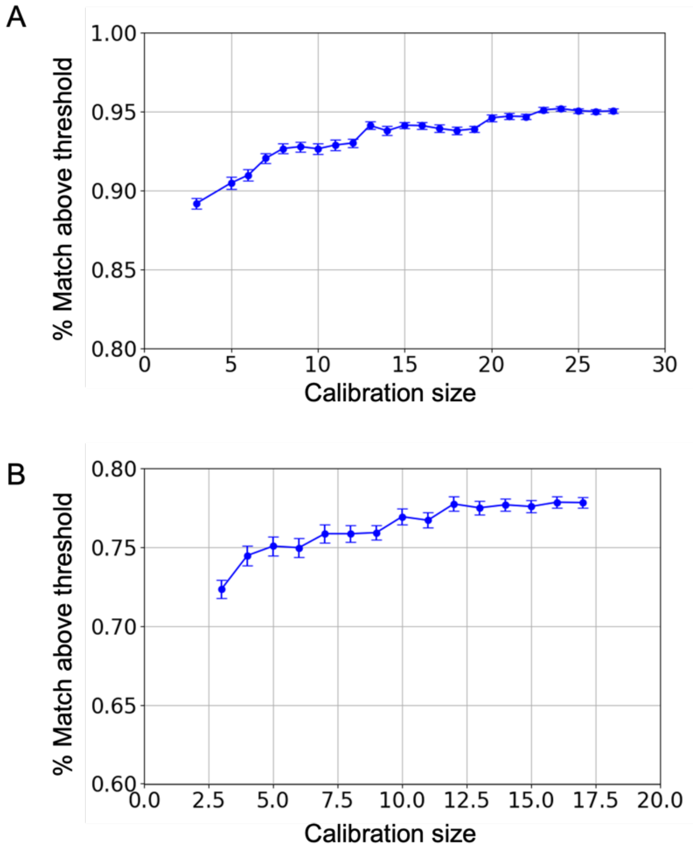

Figure S2: PlasmidCounts bootstrapping. A) Percent of calibration conditions achieving >90% match to long-read-validated plasmid counts across bootstrapped subsets of *n* genomes from a 33-genome dataset. B) Percent of calibration conditions achieving >70% match to known plasmids in simulated short-read data from 23 *E. coli* genomes. In both panels, error bars represent the standard error of the mean (SEM) across 100 parameter sets sampled from a ±75% range around default values.

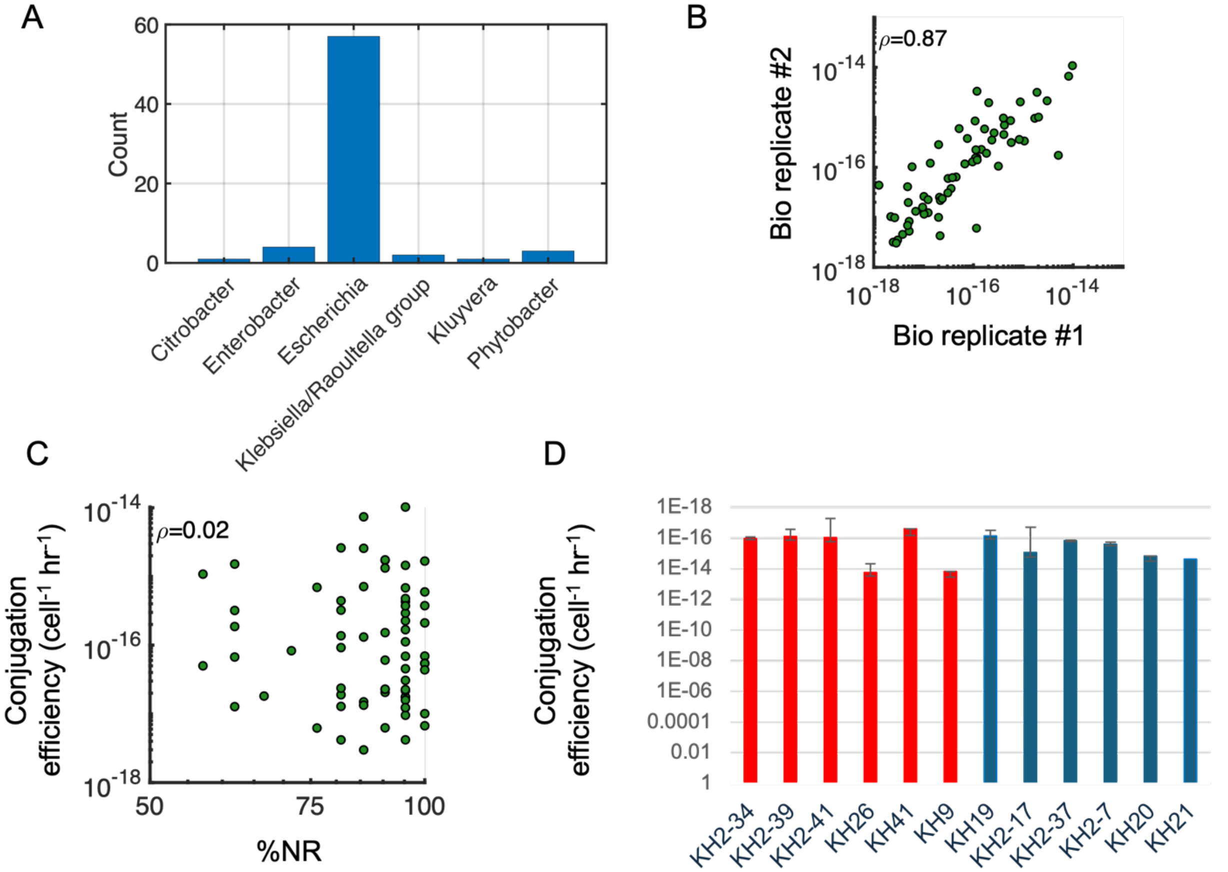

Figure S3: Conjugation efficiency supporting data. A) Distribution of species used to quantify conjugation efficiencies in Fig. 2E-F. B) Replica plot for all efficiencies, confirming that two replicates is sufficient. C) No correlation between conjugation efficiency and non-resistant percentage. D) Results are consistent with conjugation efficiencies using a second clinical plasmid, pCDC-61. Error bars indicate standard deviation of 3 biological replicates. Red bars indicate strains with 0 plasmids, and blue bars indicate strains with 3 plasmids.

Supplementary Tables:

Table S1: Dataset summary. Separate excel file containing all genomes and metadata associated with each.

Table S2: Sample Collection Information

State	City	Sampling Location	Waste Type	Number of Samples	Date Sampled
Arizona	Gilbert	Riparian Preserve	Groundwater	9	9/19/20
			Recharged Only Pond Water	9	9/19/20
			Treated Wastewater Effluent	9	9/19/20
	Gilbert	CAFO	Liquid Lagoon Manure	13	3/13/21
			Solid Lagoon Manure	1	3/13/21
			Dried Manure	4	3/13/21
	Mesa, Tempe, Guadalupe	City Sewer Pipes or Canals	Influent Wastewater	26	5/8/21, 5/11/21, 9/29/21
	Gilbert	Wastewater Treatment Plants	Influent Wastewater	4	9/29/21
	Tempe	Hospital	Influent Wastewater	4	4/22/21, 9/29/21
	Tempe	Tempe Town Lake	Surface Water	2	5/13/21
Kentucky, Washington, Illinois, New Jersey, California, Oregon	NA	Wastewater Treatment Plants	Influent Wastewater	25	9/29/21

Table S3: Geographic summary of isolates.

Location	State	Count
Barnard College, NY Campus WW	NY	100
Mesa/Tempe, AZ City WW	AZ	9
Gilbert, AZ WW	AZ	4
Guadalupe, AZ City WW	AZ	2
Washington WW Influent	WA	11
Illinois WW Influent	IL	2
New Jersey WW Influent	NJ	2
Kentucky WW Influent	KY	4
California WW Influent	CA	3
Oregon WW Influent	OR	3
Mesa/Tempe, AZ Hospital WW	AZ	2
Zinke Farm Dairy cattle manure - liquid	AZ	13
Zinke Farm Dairy cattle manure - solid	AZ	5
Riparian Preserve Groundwater	AZ	9
Riparian Preserve Recharge only	AZ	9
Mesa/Tempe Area Hospital WW	AZ	2
Mesa/Tempe Area City WW	AZ	11
Mesa/Tempe Area Campus WW	AZ	3
Mesa/Tempe Area Lake water	AZ	2
Riparian Preserve Treated WW effluent	AZ	9

Table S4: MIC reference ranges.

**Antibiotic**	**Abbreviation**	**Susceptible**	**Intermediate**	**Resistance**
Amikacin	AMI	<=16		>=64
Ampicillin	AMP	<=8	16	>=32
Ceftriaxone	AXO	<=1	2	>=4
Aztreonam	AZT	<=4	8	>=16
Cephalothin	CEP	<=8		>=16
Ciprofloxain	CIP	<=1	2	>=4
Ertapenem	ETP	<=2	1	>=8
Cefazolin	FAZ	<=16	4	>=32
Cefepime	FEP	<=4	4-8	>=16
Cefotoxin	FOX	<=8		>=32
Cefuroxime	FUR	<=4		>=32
Gentamicin	GEN	<=4	8	>=16
Meropenem	MERO	<=1	2	>=4
Piperacillin/taxobactam	P/T4	<=16/4	32/4 - 64/4	>=128/4
Cefpodoxime	POD	<=2	4	>=8
Trimethoprim/sulfamethoxazole	SXT	<=2/38		>=4/76
Ceftazidime	TAZ	<=4	8	>=16
Tetracycline	TET	<=4		>=16
Tigecycline	TGC	<=2		>=8
Ticarcillin/claculanic acid	TIM2	<=16/2		>=128/2
Tobramycin	TOB	<=4		>=16
Ampicillin/sulbactam	A/S2	<=8/4		>=32/16

Table S5: Summary of plasmids. This table contains each plasmid identified by PlasmidCounts by row, with relevant metadata including the replicon type, contig length, long-read data when applicable, and top BLAST hit.

Table S6: Accession IDs of published sequences used for PlasmidCounts verification. This table contains the compiled list of accession IDs of publicly available sequences and true plasmid counts. Shortread_genome_num is the true number of plasmids; predicted_genome_num is the predicted plasmid count from the top performing bootstrap iteration; and match_pct is the percentage of matched plasmids per strain.
